# Vaccine hesitancy among healthcare workers in low- and middle-income countries during the COVID-19 pandemic: Results from facility surveys across six countries

**DOI:** 10.1371/journal.pone.0288124

**Published:** 2023-07-07

**Authors:** Prativa Baral, Tashrik Ahmed, Pablo Amor Fernandez, Michael A. Peters, Salome Henriette Paulette Drouard, Pierre Muhoza, George Mwinnyaa, Charles Mwansambo, Charles Nzelu, Mahamadi Tassembedo, Md. Helal Uddin, Chea Sanford Wesseh, Mohamed Lamine Yansane, Julie Ruel Bergeron, Alain-Desire Karibwami, Tania Inmaculada Ortiz de Zuniga Lopez Chicheri, Munirat Iyabode Ayoka Ogunlayi, Isidore Sieleunou, Tawab Hashemi, Peter M. Hansen, Gil Shapira

**Affiliations:** 1 The World Bank, United States of America; 2 Department of International Health, Johns Hopkins Bloomberg School of Public Health, United States of America; 3 Global Financing Facility for Women, Children, and Adolescents, United States of America; 4 Ministry of Health, Malawi; 5 Federal Ministry of Health, Nigeria; 6 Ministry of Health, Burkina Faso; 7 Directorate General of Health Services, Bangladesh; 8 Ministry of Health, Liberia; 9 Ministry of Health, Guinea; Keele University School of Medicine, UNITED KINGDOM

## Abstract

**Background:**

Vaccine hesitancy remains a critical barrier in mitigating the effects of the ongoing COVID-19 pandemic. The willingness of health care workers (HCWs) to be vaccinated, and, in turn, recommend the COVID-19 vaccine for their patient population is an important strategy. This study aims to understand the uptake of COVID-19 vaccines and the reasoning for vaccine hesitancy among facility-based health care workers (HCWs) in LMICs.

**Methods:**

We conducted nationally representative phone-based rapid-cycle surveys across facilities in six LMICs to better understand COVID-19 vaccine hesitancy. We gathered data on vaccine uptake among facility managers, their perceptions of vaccine uptake and hesitancy among the HCWs operating in their facilities, and their perception of vaccine hesitancy among the patient population served by the facility.

**Results:**

1,148 unique public health facilities participated in the study, with vaccines being almost universally offered to facility-based respondents across five out of six countries. Among facility respondents who have been offered the vaccine, more than 9 in 10 survey respondents had already been vaccinated at the time of data collection. Vaccine uptake among other HCWs at the facility was similarly high. Over 90% of facilities in Bangladesh, Liberia, Malawi, and Nigeria reported that all or most staff had already received the COVID-19 vaccine when the survey was conducted. Concerns about side effects predominantly drive vaccine hesitancy in both HCWs and the patient population.

**Conclusion:**

Our findings indicate that the opportunity to get vaccinated in participating public facilities is almost universal. We find vaccine hesitancy among facility-based HCWs, as reported by respondents, to be very low. This suggests that a potentially effective effort to increase vaccine uptake equitably would be to channel promotional activities through health facilities and HCWs.However, reasons for hesitancy, even if limited, are far from uniform across countries, highlighting the need for audience-specific messaging.

## Introduction

Vaccination remains one of the most powerful, safe, and effective tools in reducing outbreaks of preventable infections and improving health outcomes worldwide, and yet, immunization programs are repeatedly confronted with various forms of hesitancy [1–3]. The phenomenon of vaccine hesitancy is defined as the “delay in acceptance or refusal of vaccines despite availability of vaccine services” [4]. According to the World Health Organization’s (WHO) SAGE Working Group on Vaccine Hesitancy, vaccine hesitancy is distributed across a complex continuum between full acceptance and outright refusal, rather than a binary construct [4]. While it is highly context-specific, and varies across time, place, and type of vaccine product, vaccine hesitancy remains a persistent and ongoing challenge globally. Well before the COVID-19 pandemic, the WHO declared in 2019 that vaccine hesitancy stands out as one of the top ten threats to global health [5]. Indeed, global trends suggest that immunization rates may stagnate due to waning vaccine confidence [6], likely contributing to the rise in vaccine-preventable diseases such as measles [7]. In low (LICs) and lower-middle-income countries (LMICs), immunization programs also face persistent barriers in accessibility to vaccines, in addition to vaccine hesitancy.

In the context of COVID-19, the global rollout of highly effective vaccines has been plagued by the lack of supply in many countries as well as delays in acceptance or refusal of the vaccine products, stemming from complex and context-specific motivations [8]. In addition to increasing supply globally, individual and community-level reasoning for hesitancy must be identified and addressed, particularly among health care workers (HCWs). HCWs are at greater risk of infection and need to be protected to ensure a health system’s functionality, especially during outbreaks. They are also regularly in close contact with vulnerable populations and patients, where vaccination can act as an important layer of protection to prevent HCW-to-patient transmission. Indeed, increased vaccine coverage against COVID-19 among health-care workers has been shown to reduce transmission of SARS-CoV-2 in hospital settings [9, 10]. Importantly, HCWs are trusted community members and play an influential role in community and patient perceptions regarding overall health and well-being–including confidence and trust in vaccines [11–13], and more broadly, public health [14]. They also play a large role in disseminating vaccine knowledge and messaging within their communities. And yet, significant vaccine hesitancy among this sub-population has been observed with vaccines against other infectious agents such as influenza [15] and continues to be prevalent in the era of COVID-19 [16]. Globally, limited evidence on the prevalence of vaccine hesitancy among HCWs exists, with some estimating the prevalence to be around 23% (range of 4.3–73%) [17], a worrying magnitude among a population that has a cascading effect in the decision-making of the public. While several studies have investigated the specific reasoning for vaccine hesitancy among this priority population in higher-income countries, a better understanding of vaccine hesitancy among HCWs in LICs and LMICs remains limited [17].

The willingness of HCWs to be vaccinated, and in turn, recommend vaccination for their patient population is an important strategy to contain the negative impacts of the COVID-19 pandemic. This cross-sectional study helps fill important gaps in available evidence by examining HCW uptake of COVID-19 vaccination in six LICs and LMICs, levels and reasons for hesitancy, and perceptions of hesitancy among the patient population attending their facilities.

## Material and methods

### Study design

Since September 2020, the Global Financial Facility (GFF) has supported 22 rounds of rapid-cycle phone-based health facility surveys across eight countries as part of the Essential Health Services Monitoring initiative [18]. These surveys were designed to understand better the constraints in maintaining essential health services, and to document adaptations to service delivery modalities. Surveys have been adapted to meet the specific needs of country partners and cover topics related to the facility’s infrastructure, finances, services, supplies, COVID-19 adaptations, and human resources for health. The facility assessments were conducted cross-sectionally, over multiple rounds, with the same sample of facilities being assessed at each round (though response rate and the respondent may vary between rounds). Implementation of each round of data collection roughly took one month, with variation depending on the country’s needs based on their epidemiological trajectory. The vaccine-hesitancy module of these rapid-cycle surveys were introduced in selected countries in 2021, for a selected period of time: this paper relies solely on the data collected during these relevant months. The survey was approved to be implemented in-country by the appropriate officials within ministries of health.

### Settings and participants

**Fig 1:**
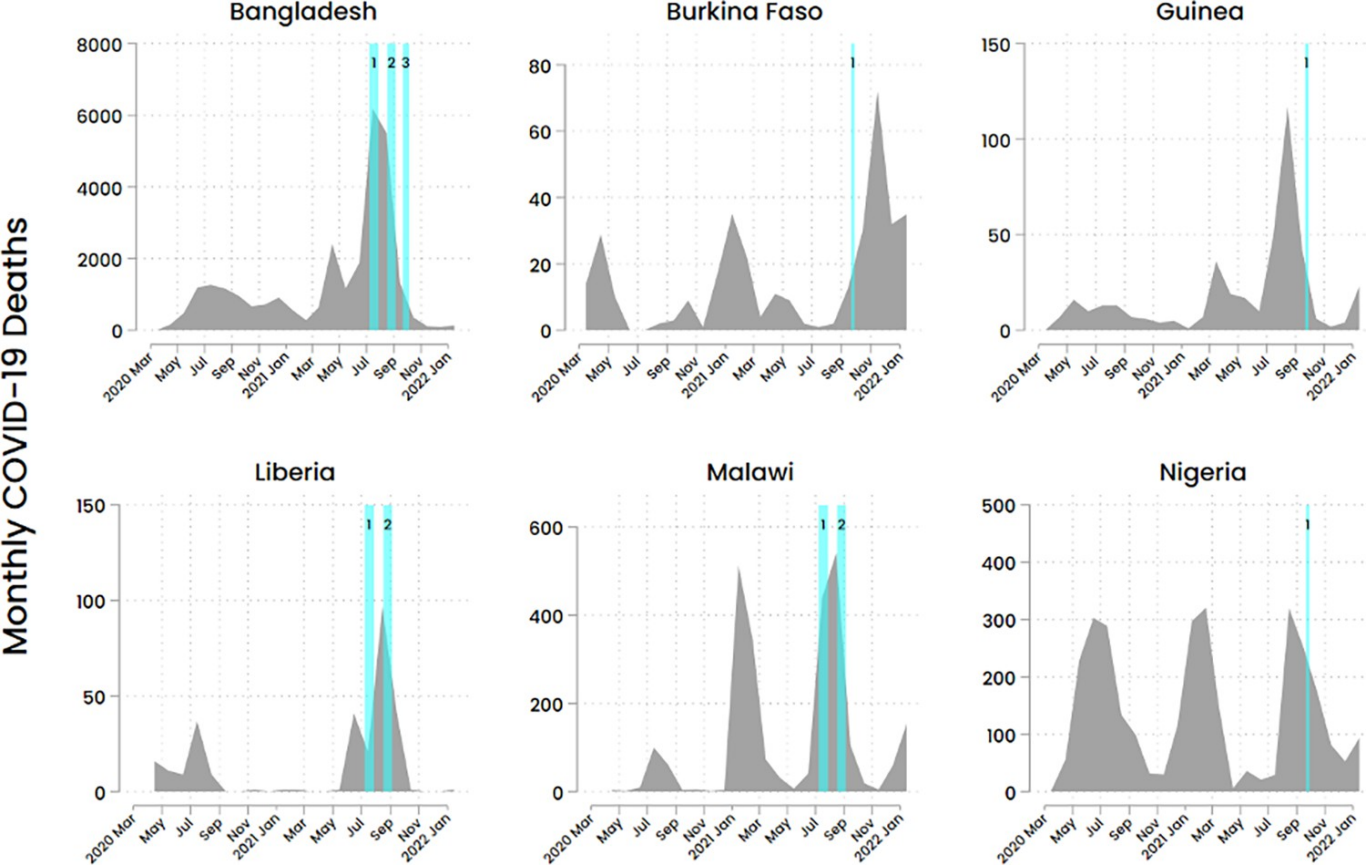
Epidemiological trajectory of the COVID-19 pandemic in participating countries*. *Epidemiological trajectory of the COVID-19 pandemic is defined by COVID-19 deaths (JHU CSSE COVID-19 Data). Blue bars denote months during which survey data were collected. Numbers within bars denote survey rounds.

This study included six countries with available and completed vaccine hesitancy modules: Bangladesh, Liberia, Nigeria, Malawi, Guinea, and Burkina Faso. See Fig 1 for graphs representing the epidemiological trajectory of the COVID-19 pandemic across the selected countries, with months during which data collection for vaccine hesitancy occurred highlighted. Survey participants included respondents from facilities, normally the head of the health facility or designate, who reported about their own perceptions, as well as their perceptions of vaccine hesitancy among HCWs in their facilities and the patient population attending their facilities. If a suitable facility representative was not available to respond, enumerators would call back later to ensure that an appropriate respondent is surveyed.

### Sampling and data collection

Public facilities within countries were selected from a master facility list provided by the corresponding Ministry of Health. Health facilities were randomly selected within each administrative unit, with the number of health facilities in the sample reflecting the weight of the stratum at the national level. Samples of the facility respondents within participating countries were all nationally representative, with the exception of Nigeria. In Nigeria, the sample of facilities was stratified by the COVID-19 burden within geopolitical zones and by facility ownership within states. Multiple rounds of data collection occurred over the course of 2021 for all six countries, though not all rounds included the vaccine hesitancy module which was introduced midway through 2021. This particular paper exclusively reports findings pertaining to the vaccine hesitancy module.

### Data collection instrument

The standard questionnaire as well as the vaccine hesitancy sub-modules were adapted to each country’s context and priorities. A sub-module was created within the larger facility phone survey tool, specifically assessing COVID-19 vaccine hesitancy among the survey respondents (a facility representative), as well as their perceptions and understanding of COVID-19 vaccine hesitancy among staff (HCWs), and the population served by the facility. Questions addressed the availability of vaccines, vaccination status of the individual respondent, their perception of vaccine uptake among HCWs, as well as reasoning for vaccine hesitancy among the unvaccinated HCWs and patient population. Vaccination status did not differentiate between receiving one dose or multiple doses. Because reasoning for vaccine hesitancy is context-specific, options for questions pertaining to reasoning were not read out loud by the enumerators to avoid any biases. Vaccination uptake was assessed through a Likert scale (“all”, “most, but not all”, “about half”, “less than half”, “none”), without quantitative references, allowing facility respondents to interpret accordingly. Cognitive probing was conducted during pilot testing to ensure that questions were easy to understand and were addressing the intended constructs [19, 20].

### Pilot testing

The survey tool was piloted in non-participating facilities within each country to verify the suitability, appropriateness, and framing of survey questions. Pilot data helped facilitate the refining of the survey instrument prior to implementation.

### Data collection procedure

Data were collected via phone surveys of participating facilities to minimize the risk of COVID-19 transmission. Respondents were informed of the objectives of the phone survey and were able to accept or decline participation. If the entirety of the survey could not be completed during one call, multiple calls were made to ensure a high response rate for all sections. Phone surveys were conducted in the country’s official language: Bengali, English, and French, and lasted no longer than 45–50 minutes on average, to minimize the burden on health care workers.

### Data analysis

Descriptive analyses using means and standard deviation of the vaccine hesitancy module were conducted in STATA 16 (Stata Corporation, College Station, TX, USA)

### Ethics and informed consent

No ethical committee review was required for Bangladesh, Guinea, Liberia, Malawi, and Nigeria, with relevant Ministries of health classifiying this work as public health practice aimed to provide immediate information to direct the country’s COVID-19 resources. For Burkina Faso, ethical clearance was obtained from the local ethics committee, Comité d’éthique pour le recherche en santé, composed of Pr. Fla Koueta, Dr. Clotaire Nanga, Pr. Maxime Drago, Pr. Kamba André-Marie Soubeiga, Mr Olivier Ouedraogo, Dr. L. Habibata Zerbo-Ouermi and Dr. Alphonse Ouedraogo. All participating respondents gave their informed consent verbally prior to answering the survey. Informed consent was documented by enumerators in written format, as a response to one of the first questions in the survey tool.

## Results

### Characteristics of facilities surveyed

Across all six countries, 1,148 unique public facilities participated in the latest round of the vaccine hesitancy module (25% Bangladesh, 14% Burkina Faso, 14% Guinea, 10% Liberia, 16% Malawi, and 21% Nigeria). (Table 1) At the time of analysis, three countries, namely Burkina Faso, Guinea, and Nigeria, had conducted one round of surveys with the vaccine hesitancy module. Bangladesh had conducted three rounds of data collection, and Liberia and Malawi had both conducted two rounds (S1 Table).

**Table 1:** Facility characteristics.

	Bangladesh (n = 286)	Burkina Faso (n = 159)	Guinea (n = 161)	Liberia (n = 118)	Malawi (n = 187)	Nigeria (n = 237)
**Location **						
*Urban (%) *	21	7	32	19	9	21
*Peri-urban (%) *	17	2	0	0	1	9
*Rural (%) *	62	91	68	81	90	70
**Facility type **						
*Hospital (%) *	33	3	3	9	3	18
*Health center (%) *	33	97	94	9	94	46
*Health Post/ Clinic (%) *	34	0	3	82	3	34
*Other (%)*	0	0	0	0	0	2

### COVID-19 vaccine hesitancy

#### Facility respondents

Findings indicate that COVID-19 vaccines have been almost universally offered to facility-based respondents (normally the head of the facility) in five of the selected countries of our study, namely Bangladesh, Burkina Faso, Guinea, Liberia, and Malawi (97–100%, with data collection occurring between end of September to early November). Respondents in Nigeria reported slightly lower availability (79%) during data collection in May 2021. Across all participating countries, when given the opportunity to get vaccinated against COVID-19, the vast majority (>94%) of survey respondents (facility representatives) reported having already received the vaccine. (Table 2) Among the very small sample of survey respondents who were offered the vaccine but had not yet received it, worries about side effects were the predominant reason for hesitancy.

**Table 2:** COVID-19 vaccine availability and uptake among survey respondents (facility representatives) in the latest round of data collection.

Country	Offered the vaccine (%)	Vaccinated (%)*
***Bangladesh*** **	97	94
***Burkina Faso***	100	95
***Guinea***	100	100
***Liberia***	100	98
***Malawi***	100	100
***Nigeria***	79	95

*Percent vaccinated among those who were offered the vaccine; we did not specify onedose or two-dose status among the vaccinated

**Numbers depict round 1 data collection; given the low sample size of unvaccinated respondents, the respondent-specific questions regarding their vaccine status were discontinued in subsequent rounds

#### Facility staff

In Bangladesh, Liberia, Malawi, and Nigeria, respondents were asked about vaccine availability for HCWs specifically. Among these four countries, almost all facility respondents from Bangladesh, Liberia and Malawi (100%, 99%, and 98% respectively) reported availability of COVID-19 vaccines for HCWs. Respondents from Nigeria reported a lower availability (76%). The survey tool in Burkina Faso and Guinea did not explicitly ask this question. Across the same four initial countries, the majority of facility respondents reported that all or most staff had also already received the COVID-19 vaccine at the time of data collection (Liberia, Malawi, Nigeria and Bangladesh >90%). The survey tool in Burkina Faso and Guinea also did not explicitly ask about uptake among HCWs (Table 3).

**Table 3:** COVID-19 vaccine availability and uptake among staff (HCWs) in the latest round of data collection.

Country	Vaccine availability (%)		Vaccinated (%)*	
	Yes	No	All staff	Most, but not all staff
***Bangladesh***	100		98	2
***Burkina Faso***	N/A**		58**	
***Guinea***	N/A**		83**	
***Liberia***	99		84	14
***Malawi***	98		56	36
***Nigeria***	76		77	20

*Percent vaccinated among health facilities that offered the vaccine

**Vaccine availability among HCWs was not explicitly asked in Burkina Faso and Guinea. In addition, uptake among HCWs in these two countries was also not explicitly asked in this round of data collection; instead, facilities reported under the “other” option for reasons for hesitancy that all of their staff had been vaccinated.

Reasons for vaccine hesitancy among HCWs, as perceived by the facility representatives responding to the survey, were numerous and varied across countries, though concerns of side effects were reported to be predominant across all countries (ranging from 34–71%, Table 4). In addition, health facility representatives in Nigeria reported that reasons for hesitancy among facility staff included belief that COVID-19 was not real (29%), a preference for natural immunity (26%) and mistrust of pharmaceutical companies and/or governments (18%). In Liberia, belief that the vaccine will infect the recipient with the COVID-19 virus (47%), and preference that others receive it first (58%) were reported to be the main concerns. Similarly, in Malawi, preference that others receive the vaccine first (24%) was cited as a concern, as well as religious beliefs contributing to hesitancy (40%). In Bangladesh, side effect concerns regarding the vaccine’s effect on reproductive and maternal health specifically were predominant (71%, though within a very small sample size of 7 facilities), as well as belief that they are medically ineligible to receive the COVID-19 vaccine (43%). Table 4 cites the main reported reasons across the six countries. S2 Table contains a comprehensive list of reasons and the respective percentages across all countries and rounds.

**Table 4:** Most common reasons for COVID-19 vaccine hesitancy among HCWs, as reported by facility representatives in the latest country-specific round of data collection*.

Bangladesh (n = 7)	Burkina Faso (n = 67)	Guinea (n = 28)	Liberia (n = 19)	Malawi (n = 82)	Nigeria (n = 38)
Worried about side effects: reproductive and maternal health (71%)	Worried about side effects (55%)	Worried about side effects (43%)	Prefer that other people get the vaccine first (58%)	Worried about side effects (63%)	Worried about side effects (34%)
Belief that they are medically ineligible to receive the COVID-19 vaccine (43%)	Mistrust of pharmaceutical companies and/or governments (34%)	Worried about side effects: reproductive and maternal health (39%)	Belief that the vaccine will infect recipient with the COVID-19 virus (47%)	Religious beliefs (40%)	Don’t believe that COVID-19 is real (29%)
N/A	Belief that they are medically ineligible to receive the COVID-19 vaccine (18%)	Mistrust of pharmaceutical companies/governments (39%)	Worried about side effects (37%)	Prefer that other people get the vaccine first (24%)	Prefer developing natural immunity to COVID-19 (26%)
N/A	Already had COVID-19 disease (15%)	Do not have adequate information about the COVID-19 (39%)	Do not have adequate information about the COVID-19 vaccine (37%)	Mistrust of pharmaceutical companies and/or governments (22%)	Mistrust of pharmaceutical companies and/or governments (18%)

*Responses reflect survey respondent perspectives on vaccine hesitancy among staff members within their facility who did not receive the COVID-19 vaccine when offered.

#### Patient population

Facility representatives were also asked about their perceptions of vaccine hesitancy among the patient population within their facilities. While the majority of survey respondents indicated that staff in Liberia, Malawi, Bangladesh, and Burkina Faso have discussed COVID-19 vaccine concerns with their patients, roughly only half of facilities in Nigeria (49%) reported having done so. In Guinea, this question was only asked when facility respondents reported availability of COVID-19 vaccines to the public; as a result, 26% of facility respondents did not respond to this question. However, among the facilities in Guinea reporting COVID-19 vaccine availability to the public, 83% indicated discussing COVID-19 vaccine concerns with their patients (Table 5). The perceptions of the facility respondents regarding vaccine hesitancy in the patient population seem to be centered predominantly around worries about side effects (55–94%) and lack of trust in government, pharmaceutical companies, or vaccines in general across all countries. Other reported concerns included belief that the COVID-19 virus is not real in Nigeria (31%), lack of trust in vaccines in Bangladesh (49%), fear that the vaccine will infect the recipient with the COVID-19 virus in Liberia (80%), and religious beliefs in Malawi (43%) (Table 6). S3 Table contains a comprehensive list of reasons across all countries and rounds.

**Table 5:** Facility representatives reporting that HCWs discussed COVID-19 vaccine concerns with patient population over multiple rounds*.

Country	*Round 1 (%)*	*Round 2 (%)*	*Round 3 (%)*
***Bangladesh***	*87*	*89*	*91*
***Burkina Faso***	*86*	*N/A*	*N/A*
***Guinea*** **	*83*	*N/A*	*N/A*
***Liberia***	*95*	*100*	*N/A*
***Malawi***	*82*	*78*	*N/A*
***Nigeria***	*49*	*N/A*	*N/A*

*As reported by facility respondents

**In Guinea, this question was only asked to facilities reporting availability of vaccines to the public

**Table 6:** Most common reasons for COVID-19 vaccine hesitancy among the patient population, as reported by facility representatives in the latest round of data collection*.

Bangladesh (n = 259)	Burkina Faso (n = 136)	Guinea (n = 67)	Liberia (n = 118)	Malawi (n = 145)	Nigeria (n = 103)
***Worried about side effects (55%)***	*Worried about side effects (82%)*	*Worried about side effects (94%)*	*Belief that the vaccine will infect recipient with the COVID-19 virus (80%)*	*Worried about side effects (83%)*	*Worried about side effects (40%)*
*Don’t trust vaccines in general (49%)*	*Do not have adequate information about the COVID-19 vaccine (43%)*	*Mistrust of pharmaceutical companies/governments (85%)*	*Worried about side effects (61%)*	*Religious beliefs (43%)*	*Don’t believe that COVID-19 is real (31%)*
*Do not have adequate information about the COVID-19 vaccine (25%)*	*Mistrust of pharmaceutical companies/governments (38%)*	*Do not have adequate information about the COVID-19 vaccine (85%)*	*Do not have adequate information about the COVID-19 vaccine (48%)*	*Mistrust of pharmaceutical companies/governments (25%)*	*Don’t trust vaccines in general (29%)*

*Responses reflect survey respondent perspectives on COVID-19 vaccine hesitancy among the patient population attending their facility

### Changes over multiple rounds (Liberia, Malawi, Bangladesh)

**Fig 2:**
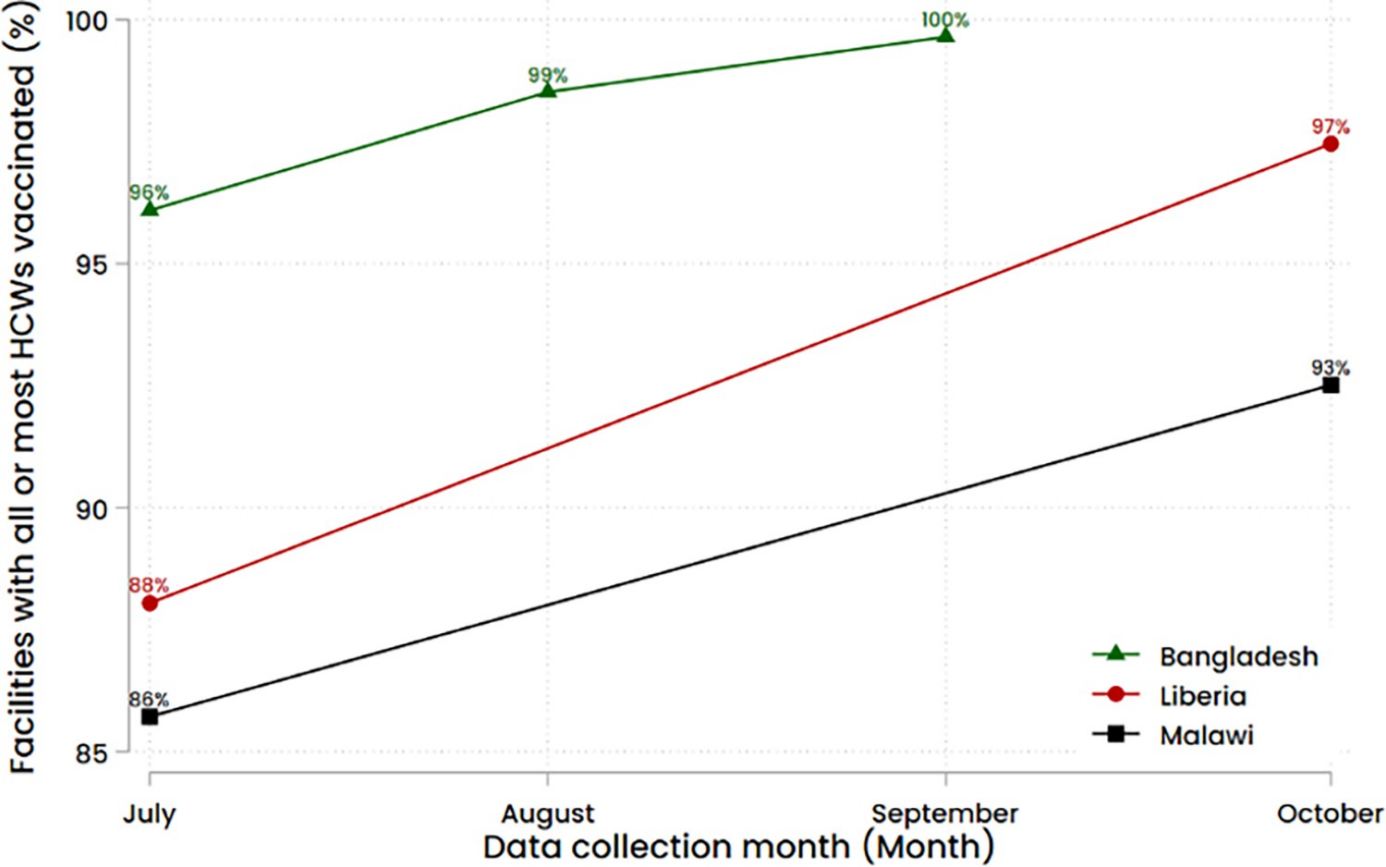
Facilities with all or most* HCWs vaccinated with the COVID-19 vaccine across multiple rounds in Bangladesh (July, August, September 2021), Malawi (July, October 2021), and Liberia (July, October 2021). *Facilities were asked about vaccination uptake among HCWs through a Likert scale (“all”, “most, but not all”, “about half”, “less than half”, “none”), without quantitative references, allowing facility respondents to interpret accordingly.

In the three countries where the vaccine hesitancy module was asked at multiple time points (Bangladesh, Malawi, and Liberia), the percentages of facilities with all or most HCWs vaccinated increased, as reported by facility representatives. Liberia reported the largest increase, from 88% to 97% of facilities (Fig 2).

## Discussion

This study contributes to the emerging understanding of COVID-19 vaccine hesitancy among HCWs. We observe low COVID-19 vaccine hesitancy among public facility-based HCWs in six countries, and an increasing percentage of vaccine uptake by HCWs in participating facilities within the three countries with multiple data points. These findings suggest that availability, rather than hesitancy, may be the primary barrier to a fully vaccinated public sector health workforce in these countries. Concern about the vaccine’s side effects was reported to be one of the top two drivers of hesitancy among HCWs in all countries. HCWs also reported that side effects were a primary reason for hesitancy in the patient population, followed by a general lack of trust in vaccines, pharmaceutical companies, and governments. As the availability of COVID-19 vaccines increases, country-specific and audience-specific reasoning for hesitancy will be critical in informing the design of vaccine campaigns and messaging, and to build overall trust, moving forward.

Despite the added risk of nosocomial transmission, previous evidence has shown a sizeable proportion of HCWs expresses COVID-19 vaccine concerns [21–23], particularly among HCWs in the African continent [24]. This is in sharp contrast to our findings, likely because our definition of HCW is restricted to facility-based medical staff, largely in upper-level facilities. While vaccine trial data has shown that severe side effects are quite rare [25, 26], further reinforcing the importance of availaibility of safety data availability, our findings suggest that among the small pool of vaccine hesitant HCWs, concerns about side effects were prevalent across the six countries. Worries about side effects may have been exacerbated by the rapid development of COVID-19 vaccines, lack of effective promotion on COVID-19 vaccine safety, and intensive media coverage of the rare but severe cases of thrombosis linked to the AstraZeneca vaccine [27]. Previous literature has indicated that potential strategies to bolster vaccine confidence among HCWs include transparency on side effects to combat mis- and disinformation [27], and appeals by vaccinated facility staff to encourage uptake among unvaccinated HCWs [22]. Given their influential role, it is vital that HCWs have access to vaccines, become vaccinated, and promote vaccination within their communities [28].

### Vaccine hesitancy among the patient population

Though our study did not specifically collect data on vaccine uptake among the patient population, it is widely recognized that in LMICs, access to COVID-19 vaccines remains a major barrier in vaccine uptake [29]. Existing literature has indicated that compared to high income countries, willingness to be vaccinated is significantly higher in LMICs [2, 30, 31]. There is nevertheless a necessity to better understand and address the nuances and geographic variations in COVID-19 vaccine hesitancy, including its association with overall trust in science and vaccines [32]. For example, social and behavioral science data by the Institute of Development Studies pointed to a high likelihood of vaccine uptake overall in the continent of Africa, with important and observable variations between countries [33]. Variations between urban and rural regions within country could also be important, though with this study, that distinction was impossible to assess given the very low rate of vaccine hesitancy reported. Our findings also suggest that HCWs perceive the concerns about vaccine side effects to be the most common reason for hesitancy among the patient population they serve. This is consistent with findings from previous studies [2], as well as the World Bank’s COVID-19 Household Monitoring Dashboard [34]. As access to COVID-19 vaccines becomes less of a barrier to vaccination, it is imperative that appropriate context-specific risk messaging, ideally via HCWs, be implemented to minimize the burden of COVID-19 in these communities.

### Differences between staff and patient population, as perceived by facility respondents

Though the small sample size for vaccine hesitancy reasoning limited statistical comparison between sub-populations, concerns regarding vaccine side effects were cited consistently across both staff and patient populations in all countries. In addition, issues of scientific and governmental trust may be contributing to vaccine hesitancy, particularly in the patient population. These included not trusting vaccines in general (Bangladesh and Nigeria), mistrust of pharmaceutical companies and governments (Burkina Faso, Guinea, and Malawi), as well as the belief that the vaccine contained the COVID-19 virus and will infect the recipient (Liberia). Previous studies outside of the context of COVID-19 have highlighted this concern [35, 36], given negative experiences historically with foreign entities using vaccination campaigns for other motives [37], as well as doubt and mistrust in a government’s capacity and integrity to handle a public health crisis [38]. As vaccines become increasingly available, trust is likely to evolve and will need to be monitored closely given its significant implications in a country’s ability to increase vaccine acceptance, not just with COVID-19, but with other crises as well [39–41]. Additional assessments on the role of HCWs in building trust would also be beneficial.

### Changes over multiple rounds (Liberia, Malawi, Bangladesh)

Among the countries with multiple rounds of data collection, findings indicate an overall trend towards an increasing vaccine uptake among HCWs, as perceived by survey respondents. Clear discrepancy in reasoning for hesitancy in the increasingly small pool nevertheless remained across settings. For example, lack of information about vaccines and misinformation (e.g., vaccines will result in deaths in 2 years) was reported to be one of the larger sources of hesitancy in Liberia, whereas religious beliefs appeared important in Malawi. In Bangladesh, perceived ineligibility of vaccines was observed to be an important source of hesitation. This trend in increasing uptake needs to be similarly assessed in other countries to confirm this finding. Findings suggest that vaccine hesitancy is not static and beliefs change over time, and can be influenced.

### Limitations

Our study includes several limitations. First, given the methodology used for data collection via phone-based facility surveys, responses were attributed by one individual acting as the facility representative, to speak on behalf of the entirety of the staff and patient population. Because the representative often held a senior role in most facilities, they may have a different reasoning process from more junior staff, which limits opportunities for generalizability of findings. This also restricts representativeness to sub-populations that work or seek care in formal public health facilities, as opposed to also including informal or private sectors. We also could not stratify hesitancy by individual characteristics, given the design of the study and the survey tool. However, the large sample of HCWs nevertheless provides insights from multiple countries. Second, the data cannot be extrapolated more generally to LMICs given that reasons for vaccine hesitancy are context specific. Nevertheless, our study does include insights from three continents, enabling a global perspective. Third, the timing of the phone survey may have impacted responses depending on the prevalence of COVID-19 and/or access to vaccines at the time of the survey. Because there was variation in the timing of the survey and data collection, the epidemiological trajectories of participating countries, access to COVID-19 vaccines, as well as other possible external factors at play during an evolving pandemic, estimates may not necessarily be comparable across different countries and contexts. However, the aim of the study was to provide multiple snapshots or estimates of vaccine hesitancy across multiple rounds using descriptive data, rather than provide a direct comparison (impossible, given external factors listed). Fourth, the interpretability of vaccine hesitancy and its reasoning may be subject to context: side effects can be understood differently by different enumerators in different communities.

Despite these limitations, data collected from the perspective of a health facility respondent can provide a general status of vaccine hesitancy within their facility, which can be helpful as a first step towards understanding vaccine coverage and uptake within a community. In addition, understanding how HCWs are interpreting vaccine hesitancy as a problem, and their perceptions of drivers of hesitancy among staff and patient populations, can be helpful in effectively tailoring vaccine messaging to specific audiences. Given the limited resources and constraints during a pandemic, implementing individual surveys on vaccine hesitancy may not always be feasible. Collecting data on vaccine hesitancy using phone-based health facility surveys can be an efficient approach—due to low cost, relatively short duration, and with rapid turnaround time—to rapidly monitor coverage and hesitancy within key populations. These exploratory assessments can then serve as an entry point for deeper quantitative and qualitative studies that fill the relevant knowledge gaps.

### Policy implications

Our findings point to high vaccine uptake and lower hesitancy among HCWs when vaccines are made available. A potentially effective intervention to combat vaccine hesitancy is to involve HCWs in promoting vaccines to their patient population, and more broadly, to the public. Additional setting-specific research is also required to better understand the environment and existing barriers faced by the patient population. As access to COVID-19 vaccines becomes less of a barrier, our study’s reported low rates of vaccine hesitancy among HCWs indicate that national immunization campaigns in LICs and LMICs should focus on influencing the positive intentions among the patient and general population towards action, namely vaccine uptake. Developing flexible and rapid context-specific communication strategies (e.g., phone calls, SMS text messages, television, radio, social media, posters) to reach vast segments of the population should be emphasized. In addition, given the variability in knowledge, perceptions, attitudes and beliefs around vaccines and the resulting complexity in vaccination-related decision-making, our findings suggest that a tailored approach to messaging would be most beneficial. More broadly, there is also the need for countries to invest and strengthen infrastructure and capacity for monitoring public perception and responding to dis- and mis-information, as well as investing in COVID-19 and general vaccine safety surveillance and pharmacovigilance. These efforts would support ongoing surveillance by individual manufactuers and enable MoHs to address country contexts using locally-relevant data.

Finally, this discourse should include considerations of vaccine equity, and the moral, ethical, and scientific need to share doses globally. Given the high uptake among HCWs demonstrated by our findings, prioritizing distribution to LICs and LMICs—with clear channels of distribution within country—can increase global vaccination coverage. This, in turn, will reduce opportunities for COVID-19 to spread and mutate, bringing the world closer to the end of the pandemic. Efforts to address barriers to action should therefore prioritize improving vaccine access, in addition to combatting vaccine hesitancy.

## Supporting information

S1 Table Number of participating facilities with multiple rounds of vaccine hesitancy data collection. (XLSX)

S2 Table Reasoning for COVID-19 vaccine hesitancy among HCWs across countries and rounds of data collection, as reported by facility respondents. (XLSX)

S3 Table Reasoning for COVID-19 vaccine hesitancy among the patient population across countries and rounds of data collection, as reported by facility respondents. (XLSX)
